# Volatiles Profiling, Allelopathic Activity, and Antioxidant Potentiality of *Xanthium Strumarium* Leaves Essential Oil from Egypt: Evidence from Chemometrics Analysis

**DOI:** 10.3390/molecules24030584

**Published:** 2019-02-07

**Authors:** Ahmed Abd El-Gawad, Abdelsamed Elshamy, Abd El-Nasser El Gendy, Ahmed Gaara, Abdulaziz Assaeed

**Affiliations:** 1 Plant Production Department, College of Food & Agriculture Sciences, King Saud University, Riyadh 11451, Saudi Arabia; assaeed@ksu.edu.sa; 2 Department of Botany, Faculty of Science, Mansoura University, Mansoura 35516, Egypt; 3 Department of Natural Compounds Chemistry, National Research Centre, 33 El Bohouth St., Dokki, Giza 12622, Egypt; elshamynrc@yahoo.com (A.E.); ahmedgaara2003@live.com (A.G.); 4 Faculty of Pharmaceutical Sciences, Tokushima Bunri University, Yamashiro-cho, Tokushima 770-8514, Japan; 5 Medicinal and Aromatic Plants Research Department, National Research Centre, 33 El Bohouth St., Dokki, Giza 12622, Egypt; aggundy_5@yahoo.com

**Keywords:** *Xanthium strumarium*, essential oil, PCA, AHC, phytotoxicity, antioxidant activity

## Abstract

The essential oil (EO) of *Xanthium strumarium* L. leaves (family: Asteraceae) was extracted by hydrodistillation, and then analyzed by gas chromatography-mass spectrometry (GC-MS). Forty-three essential compounds were identified. The sesquiterpenoids represented the major constituents (72.4%), including oxygenated (61.78%) and non-oxygenated (10.62%) sesquiterpenes, followed by monoterpenes (25.19%). The diterpenoids and oxygenated hydrocarbons were determined as minor compounds. The main constituents of the EO were 1,5-dimethyltetralin (14.27%), eudesmol (10.60%), l-borneol (6.59%), ledene alcohol (6.46%), (-)-caryophyllene oxide (5.36%), isolongifolene, 7,8-dehydro-8a-hydroxy (5.06%), L-bornyl acetate (3.77%), and aristolene epoxide (3.58%). A comparative analysis was stated here between the EO of Egyptian *X. strumarium* and those previously reported from Pakistan, Iran, and Brazil based on chemometic tools such as principal components analysis (PCA) and agglomerative hierarchical clustering (AHC). The EO of *X. strumarium* showed weak 1, 1-diphenyl-2-picryl hydrazyl (DPPH) radical scavenging activity with IC_50_ 321.93 µL*/*L^−1^, which was comparable to ascorbic acid as a reference. However, the EO exhibited significant allelopathic potential regarding the germination and growth of the noxious weed *Bidens pilosa* in a concentration-dependent manner. Therefore, further study is recommended to characterize the EO from *X. strumarium* as an eco-friendly green bioherbicide against weeds, as well as determine their mode of actions.

## 1. Introduction

From ancient times, the essential oils (EOs) of herbal plants and other plant extracts have attracted the attention and interest of researchers as sources of bioactive natural products. EOs were reported to exhibit several significant biological activities as antioxidant and antimicrobial agents as well as have the potential for food preservation [1,2]. The free radicals play a critical role in human health in relation to oxidative stress; they damage the cell components, resulting in cellular and metabolic injury in the form of inflammation, as well as cardiovascular and cancer diseases [2].

On the other hand, using synthetic herbicides has exhibited harmful effects on the environment, and therefore human health, in addition to the appearance of herbicide-resistant weeds [3]. Nowadays, allelopathy from different parts of plant materials offers significant sources for selective biological weed management through the release and production of allelochemicals [4]. The EOs produced from different plant parts provide an ecological advantage such as protection against predators, determinants of vegetation patterning, pollinator attractants, and the mediation of plant–plant interactions, including allelopathy [5,6,7]. EOs have been recommended as potent agents for retarding plant growth and inhibiting seed germination [8,9].

The species belonging to *Xanthium* genus (family: Asteraceae) were reported to exhibit several bioactivities such as antimalarial [10], cytotoxicity [11], insecticidal [12], fungicidal [13], antiviral [14], and antibacterial [15]. The annual herbal plant *X. strumarium* L. is widely distributed around the world, especially in Africa, Asia, and Europe. *Xanthium strumarium* (Cocklebur) was used in folklore medicine for the treatment of headache, arthritis, urticaria, emphysema, sinusitis, diuretic, sedative, and diaphoretic ailments [16,17,18]. This plant is used in the manufacturing of yellow dye, which is used as hair dye; from this, the genus took the name, as xanthos means yellow in Greek [19]. The chemical constituents of *X. strumarium* are enriched with phenolics [16], steroids, triterpenoid saponins [20], monoterpene, sesquiterpene [21,22], and EO [23,24,25].

According to our knowledge, no study has yet revealed the composition of EOs from Egypt. Herein, the goals of our study were (i) identification of the chemical constituents of the EO of *X. strumarium* leaves collected from Egypt, (ii) comparison the EO of Egyptian *X. strumarium* with those previously documented from Pakistan, Iran, and Brazil, depending upon principal components analysis (PCA) and agglomerative hierarchical clustering (AHC), (iii) evaluation of the allelopathic potential of the EO on the germination and growth of the noxious weed *Bidens pilosa* and (iv) assessment of antioxidant activity of the EO.

## 2. Results and Discussion

### 2.1. Chemical Constituents of EO of the Leaves of *X. Strumarium*

**Figure 1:**
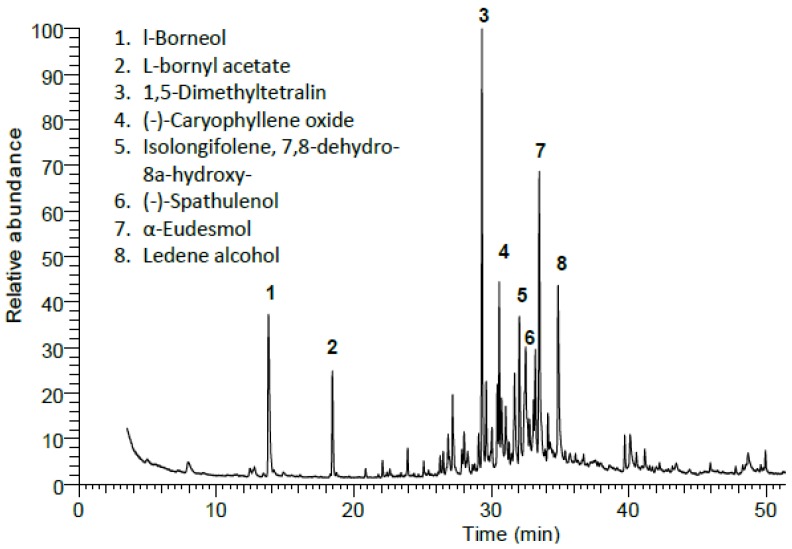
Gas chromatography-mass spectrometry (GC-MS) chromatogram of essential oil (EO) from the leaves of Egyptian *X. strumarium*. The peaks with numbers represent the major compounds.

Forty-three compounds were identified, which represented 100% of the total mass (Figure 1 and Table 1). Four classes of organic essential components (monoterpenes, sesquiterpenes, diterpenes, and oxygenated hydrocarbons) were identified.

The gas chromatography-mass spectrometry (GC-MS) of the EO exhibited that this plant is very enriched with monoterpenoids (25.19%), including both non-oxygenated (14.27%) and oxygenated (10.92%) types. Non-oxygenated monoterpenes are represented by only one compound (1,5-dimethyltetralin). However, the oxygenated monoterpenes were represented by l-borneol (6.59%) and L-bornyl acetate (3.77%) as majors, while α-terpinyl acetate (0.56%) was detected as a minor.

The sesquiterpenes represented the major components by a ratio of 72.40%. The identified sesquiterpenoids were classified into two classes, including oxygenated and non-oxygenated types with a ratio of 61.78% and 10.62% respectively. The major constituents in the identified non-oxygenated sesquiterpenes were *β*-cadinene (1.36%), *α*-calacorene (1.34%), and *β*-selinene (1.16%), but *γ*-himachalene (0.29%) was identified as a minor one. However, eudesmol (10.60%), ledene alcohol (6.46%), (-)-caryophyllene oxide (5.36%), and isolongifolene 7,8-dehydro-8a-hydroxy (5.06%) represented the main components of the oxygenated sesquiterpenes. Also, aristolene epoxide (3.58%), calarene epoxide (3.52%), and aristolone (2.84%) were identified with high ratios from the total mass of oxygenated sesquiterpenes, while 9-methoxycalamenene (0.22%) represented the minor component.

Diterpenoids represented only 0.78% of the total mass, with only two identified compounds, which were characterized as phytol acetate (0.49%) and (8β,13β)-kaur-16-ene (0.29%). Overall, the GC-MS results exhibited that the leaves of *X. strumarium* are very enriched with terpenoids by 98.37% of the total mass.

The oxygenated hydrocarbons with a small ratio of 3.33% of the total mass were also characterized. Only four compounds were identified as oxygenated hydrocarbons, including 6,10,14-trimethyl-2-pentadecanone (1.23%) as a major, while cis-13,16-docasadienoic acid (0.52%) was detected as a minor compound.

The chemical composition of EOs is complex and very rich with several bioactive substances [26] such as monoterpenes, sesquiterpenes, diterpenes, hydrocarbons, and their aldehyde and phenolic derivatives. The concentrations and chemical constituents of EOs of the aromatic herbal plants depended upon several environmental factors such as plant species, geographical sources, soil, climatic conditions, weather, seasons of the year, and vegetative cycle stages [27,28]. Also in plant species, a strong direct relation was deduced between the EOs and the pollination, seeds dissemination, and defense routes against attacks of herbivores as well as microorganisms such as fungi and bacteria [29,30].

**Figure 2:**
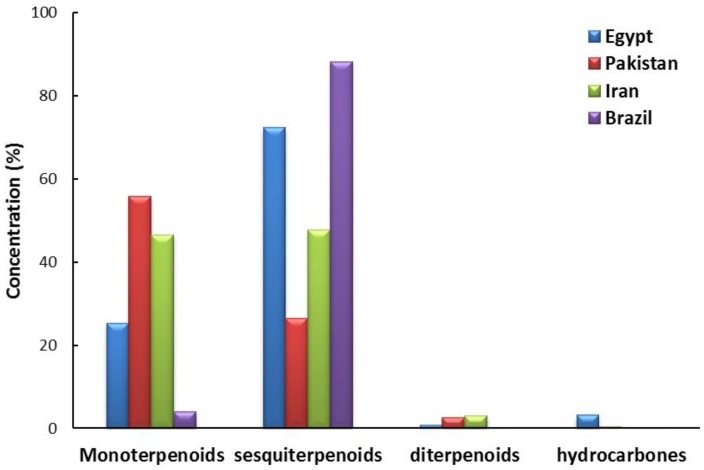
Percentage of various classes of the identified components in EOs from the leaves of *X. strumarium* from Egypt, as well as those reported from Pakistan [24], Iran [23,25], and Brazil [31].

Several reports described the constituents and biological activities of EO from the leaves of *X. strumarium* collected from Pakistan [24], Iran [23,25], and Brazil [31]. In harmony with our study, all of them are very commonly enriched with different classes of essential compounds, especially monoterpenes and sesquiterpenes. According to these previous studies, we described here for the first time the chemical constituents of the leaves of *X. strumarium* collected from Egypt. Our findings were in agreement with those previously reported studies (Figure 2). Esmaeili et al. [23] and Sharifi-Rad et al. [25] reported that the EO from leaves of Iranian (Iran-Sistan and Iran-Lurestan) *X. strumarium* are enriched with mono and sesquiterpenes as two main classes of components by 46.60% and 47.81%, respectively, in addition of diterpenes and hydrocarbons as minors. Parveen et al. [24] described that the monoterpenoids are main components (55.80%), followed by sesquiterpenes (26.40%), in addition to diterpenes and hydrocarbons as minors in the EO from leaves of *X. strumarium* collected from Pakistan. However, the EO of leaves of Brazilian *X. strumarium* was stated to include the sesquiterpenes as main components (88.13%) in addition to monoterpenes (4.00%) without any diterpenes and hydrocarbons [31]. However, our study exhibited that the EO of leaves of Egyptian *X. strumarium* were close to the EO of Brazilian ecospecies, in which the sesquiterpenes are the main components (72.40%). Meanwhile, our findings deduced that the Egyptian species are similar to the Pakistani and Iranian species in which the monoterpenes (25.19%) represent the second major components, followed by the minors of hydrocarbons and diterpenes (Figure 2).

In conclusion of our chemical study, the variations of the chemical composition of extracted EOs from plant species often occur, since the production of metabolites, involving EOs, is substantially influenced by environmental factors such as seasonality, age, plant development, circadian rhythm, temperature, water availability, altitude, nutrients, atmospheric composition, the different plant organs, and attack of pathogens and herbivores [32,33].

### 2.2. Principal Components Analysis (PCA) and Agglomerative Hierarchical Clustering (AHC)

**Figure 3:**
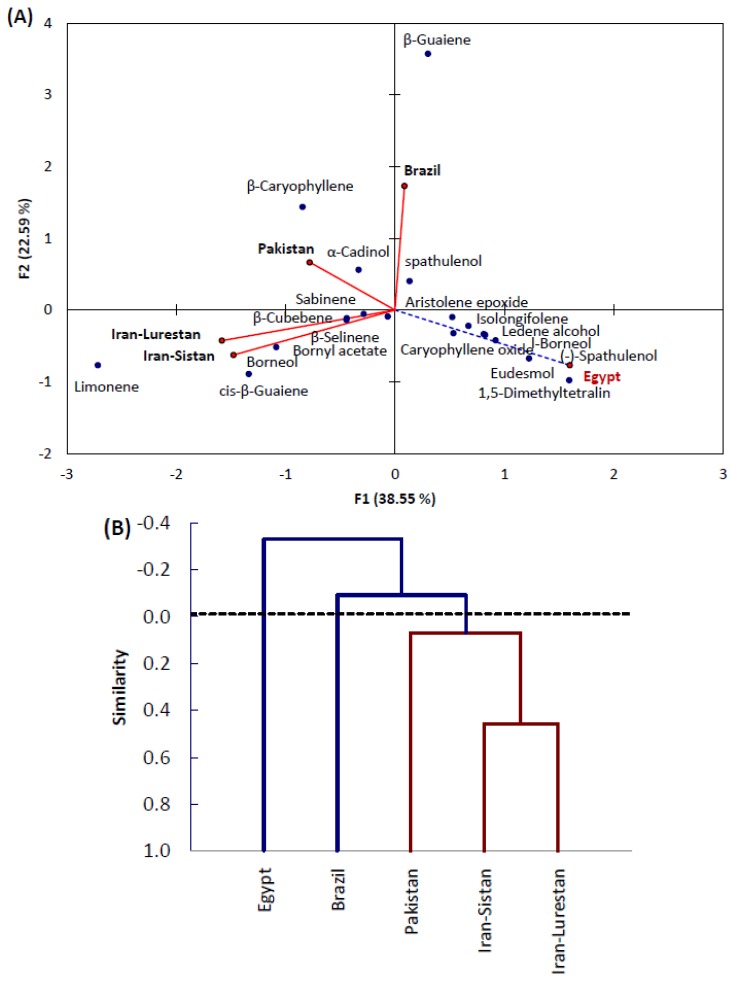
(**A**) Principal component analysis (PCA) and (**B**) agglomerative hierarchical clustering (AHC) based on the chemical composition of the EO derived from Egyptian ecospecies of *X. strumarium* leaves as well as Pakistani, Iranian, and Brazilian ecospecies.

In order to assess the correlation between the Egyptian ecospecies as well as Pakistani [24], Iranian [23,25], and Brazilian [31] ecospecies, the GC-MS data of the major compounds of the EO was subjected to PCA. The PCA horizontal axis explained 38.55% of the total variance, while the vertical axis explained a further 22.59% (Figure 3A). It is clear that both Iranian ecospecies (Iran-Sistan and Iran-Lurestan) are significantly correlated with each other as well, and are also correlated with the Pakistani ecospecies (Figure 3A and Table 2). However, the Egyptian ecospecies did not show any correlation with all of the other ecospecies.

The major compounds of the Egyptian ecospecies (1,5-dimethyltetralin, eudesmol, (-)-spathulenol, l-borneol, ledene alcohol, caryophyllene oxide, isolongifolene, 7,8-dehydro-8a-hydrox-, and aristolene epoxide) had high positive loading on PC1. However, spathulenol seems to be more correlated with the Pakistani ecospecies than the Egyptian species.

On the other hand, both Iranian ecospecies showed significant correlation with borneol, bornyl acetate, cis-*β*-guaiene, limonene, sabinene, *α*-cadinol, and *β*-cubebene. Meanwhile, the Pakistani ecospecies seemed to be more correlated with the Iranian ecospecies, where they were correlated through limonene. However, Pakistani showed a close correlation to *β*-caryophyllene and *α*-cadinol. This correlation between these two ecospecies could be ascribed to the similarity of the geographical region’s environmental and climatic conditions, as well as the genetic relation [34,35]. The Brazilian ecospecies did not show any correlation with other ecospecies, while it showed a close correlation with *β*-guaiene.

Agglomerative hierarchical clustering (AHC) based on the similarity led to the recognition of three groups (Figure 3B). Group I comprised both the Iranian ecospecies (Iran-Sistan and Iran-Lurestan) and Pakistani ecospecies, group II contained the Brazilian ecospecies, and finally group III comprised the Egyptian ecospecies. Generally, we can say that the chemical composition of the EO from the *X. strumarium* leaves of Egyptian ecospecies is completely not correlated to other ecospecies. This could be attributed to the variation in climatic conditions, environmental factors, the age of the plant, and extraction techniques [36].

### 2.3. Allelopathic Activity

*X. strumarium* EO showed a significant allelopathic activity on the germination and growth of the noxious weed *B. pilosa* in a concentration-dependent manner (Table 3). At higher concentrations (1000 μL L^-1^), the germination of seeds, root, and shoot growth were inhibited by 97.34%, 98.45%, and 93.56%, respectively. This strong inhibition could be attributed to the major compounds reported in the EO such as eudesmol, (-)-spathulenol, l-borneol, ledene alcohol, caryophyllene oxide, isolongifolene, 7,8-dehydro-8a-hydroxy, and aristolene epoxide, where these compounds may act either singular or synergetic as allelopathic agents against weeds. These are mainly oxygenated sesquiterpenes where they were reported to have various biological activities, including allelopathic activity [37,38].

The sesquiterpene *α*-eudesmol has wide interesting bioactivity, such as anticancer, antifeedant, plant growth regulation [39], and antimicrobial activity [40]. Moreover, Abd El-Gawad et al. [41] refered to the allelopathic potential of *Rhynchosia minima* to the high concentration of eudesmol. In addition, borneol [42] and caryophyllene oxide [7] were also reported to possess allelopathic activity.

*B. pilosa* is a noxious weed species, where it reduces the yield in several crops due to its fast growth and potential competitive activity [43]. It is worth mentioning here that the EO from *X. strumarium* in the present study revealed double fold the allelopathic activity against *B. pilosa* than those from *Cullen plicata* [7]. Therefore, we can say that the *X. strumarium* EO could be considered as a potential green source for bioherbicides, at least against the noxious weed *B. pilosa*; it can also be integrated into the eco-friendly weed control strategies. However, further investigation is needed in order to characterize its mode of actions, as well as the biosafety in the application at the field level.

### 2.4. Antioxidant Activity

The antibacterial, antifungal, and scolicidal activity EO of the leaves of *X. strumarium* collected from Pakistan [24] and Iran [23,25] were evaluated. Herein, the DPPH radical scavenging activity of EO from leaves of Egyptian ecospecies of *X. strumarium* was evaluated for the first time.

In our study, the capabilities of *X. strumarium* essential oil to donate hydrogen atoms or electrons were evaluated spectrophotometrically. The ability of the tested oil to possess antioxidant activity by the reduction of DPPH to diphenyl-picrylhydrazine (the yellow-colored product), and decreasing the absorbance at 517 nm comparable to ascorbic acid as a reference antioxidant agent. Our evaluation result exhibited that the EO of *X. strumarium* exhibited weak DPPH radical scavenging activities with IC_50_ 321.93 µL L^−1^ by comparable to 35.07 µL L^-1^ for ascorbic acid (Table 4).

Mata et al. [44] reported that the weak DPPH radical scavenging activity of the terpene components can be attributed to them not having the ability to donate hydrogen atoms and their low solubility in the medium of the assay. Thus, the EO of *X. strumarium* exhibiting weak activity can be attributed to the main constituents of the oil being almost monoterpene and sesquiterpene hydrocarbons. According to Viuda-Martos et al. [45] and Andrade et al. [32], these previous factors may be the main limitation of measuring the DPPH radical scavenging activity of lipophilic samples, similar to many essential oils. In addition, Ruberto and Baratta [46], the presence of methylene group molecules may play an important role in increasing the antioxidant behavior. So, the weak antioxidant activity of EO of *X. strumarium* is attributed to the major compounds in this oil being monoterpenes and sesquiterpenes hydrocarbons and/or substituted oxygenated ones. Our finding is consistent with that reported from the EO extracted from the Brazilian ecospecies of *X. strumarium* [31].

## 3. Material and Methods

### 3.1. Plant Material

The leaves of *X. strumarium* were collected in the flowering stage in March 2017 from El-Salhia region, Al-Sharkia Province, Egypt (30°38′59″N 31°57′53″E). The collected plant was identified by one from the author, Associate Professor Ahmed M. Abd El-Gawad. A voucher specimen (Mans.001024019) was deposited in the herbarium of Botany Department Faculty of Science, Mansoura University, Egypt.

### 3.2. Extraction of EO

The EO of the fresh leaves (300 g) of *X. strumarium* (0.021 mL) was extracted by hydrodistillation using a Clevenger-type apparatus for three hours. The oily layer was separated using diethyl ether and dried with anhydrous sodium sulfate (0.5 g). This extraction was repeated two times, which afforded two samples of EO. The extracted two samples of EO were stored in sealed air-tight glass vials at 4 ºC until further analysis.

The EO components of the two extracted samples were analyzed separately and identified depending upon GC-MS analysis. The GC-MS analysis of the EO samples were carried out using gas chromatography-mass spectrometry instrument stands at the Department of Medicinal and Aromatic Plants Research, National Research Center, Dokki, Giza, Egypt with the following specifications, Instrument: a TRACE GC Ultra Gas Chromatographs (THERMO Scientific Corp., Miami, CA, USA), coupled with a thermo mass spectrometer detector (ISQ Single Quadrupole Mass Spectrometer; Model ISQ spectrometer, THERMO Scientific Corp. ). The GC-MS system was equipped with a TR-5 MS column (30 m × 0.32 mmi.d., 0.25-μm film thickness, THERMO Scientific Corp. ). Analyses were carried out using helium as a carrier gas at a flow rate of 1.0 mL min^−1^ and a split ratio of 1:10 using the following temperature program: 60 °C for one minute; rising at 4.0 ^o^C min^−1^ to 240 °C, and held for one minute. The injector and detector were held at 210 °C. Diluted samples (1:10 hexane, *v*/*v*) of one μL of the mixtures were always injected. Mass spectra were obtained by electron ionization (EI) at 70 eV, using a spectral range of *m/z* 40–450.

### 3.3. Identification of EO Constituents

The identification of the chemical constituents of the EO was deconvoluted using AMDIS software (NIST, Gaithersburg, MD, USA; Wiley, Hoboken, NJ, USA) and identified by its retention indices (relative to *n*-alkanes C_8_-C_22_), mass spectrum matching to authentic standards (when available), the Wiley spectral library collection, and the NSIT library database.

### 3.4. Allelopathic Activity

The leaves of *X. strumarium* were collected from different orchards cultivated with mango trees at El-Salhia region, Al-Sharkia Governorate, Egypt (30°38′59″N 31°57′53″E). The collected samples were cleaned from dust, dried in a shaded place at room temperature (27 °C ± 2), ground, and packed in a paper bag until further use. However, the seeds of *Bidens pilosa* were collected from a garden of Mansoura University, Mansoura, Egypt (31°02′40.9″N 31°21′17.8″E).

In order to assess the allelopathic potential of the EO, various concentrations (250 µL L^-1^, 500 µL L^−1^, 750 µL L^−1^, and 1000 µL L^−1^) of the extracted EO were prepared using Tween^®^80 (Sigma-Aldrich, Germany), at the ratio 1:1 (*v*/*v*). The seeds were sterilized using 0.3% sodium hypochlorite for three minutes, followed by washing with distilled water and drying over sterilized filter paper. Subsequently, 20 seeds were transferred to sterilized Petri plates (nine centimeters) lined with two layers of Whatman No. 1 filter papers. An aliquot of four mL of either the EOs or Tween as control was added to the plates and sealed with a Parafilm^®^ tape (Sigma, USA) and kept at 27°C in a growth chamber (Abd El-Gawad, 2016).

After five days of incubation, the germinated seeds were counted, and the length of the root and shoot were measured. The inhibition of germination or seedling length was calculated as follows:

(1) $$\mathrm{Inhibition}\;\left(\%\right)=100\times \frac{\left(\mathrm{No}/\mathrm{Length}\;\mathrm{of}\;\mathrm{control}-\mathrm{No}/\mathrm{Length}\;\mathrm{of}\;\mathrm{tretamnet}\right)}{\mathrm{No}/\mathrm{Length}\;\mathrm{of}\;\mathrm{control}}$$

### 3.5. Antioxidant Activity

The antioxidant activity of the EO was measured based on the radical scavenging activity of the stable radical, 2,2-diphenyl-1-picrylhydrazyl (DPPH) (Sigma-Aldrich, Darmstadt, Germany) according to the methods of Miguel [47]. A reaction mixture of one mL of different concentrations of the EO and an equal volume of the alcoholic solution of 0.3 mM of DPPH were prepared, mixed well, and incubated in the dark for 15 minutes at room temperature (25 °C). In addition, various concentrations of ascorbic (standard antioxidant) were subjected to the same procedures. The absorbance at 517 nm was determined using a spectrophotometer (Spectronic 21D model, Milton Roy, CA, USA). The IC_50_, which is the amount of material necessary to decrease the absorbance of DPPH by 50%, was calculated graphically.

### 3.6. Statistical Analysis

The data of both allelopathy and antioxidant, in triplicates, were subjected to one-way ANOVA followed by Duncan’s test at a probability level of 0.05 using the COSTAT software program. However, the data derived from the GC-MS analysis of Egyptian ecospecies and of other reported ecospecies (Pakistani, Iranian, and Brazilian) were subjected to an agglomerative hierarchical cluster (AHC) based on 80 identified chemical compounds. We also constructed a matrix of correlation by a principal component analysis (PCA) to identify whether a significant difference exists between different ecospecies. Both AHC and PCA were performed using XLSTAT statistical computer software package, version 14 (Addinsoft, New York, NY, USA).

## 4. Conclusions

The present study revealed that the chemical composition of the EO from the leaves of *X. strumarium*, an Egyptian ecospecies, was different from other ecospecies reported from Pakistan, Iran, and Brazil. This difference could be attributed to the variation in climatic and environmental conditions. The Egyptian *X. strumarium* ecospecies EO contained 43 compounds with major constituents related to sesquiterpenoids. The main constituents were 1,5-dimethyltetralin, eudesmol, l-borneol, ledene alcohol, (-)-caryophyllene oxide, isolongifolene, 7,8-dehydro-8a-hydroxy, L-bornyl acetate, and aristolene epoxide. The EO showed weak antioxidant activity. However, the EO showed strong allelopathic activity against the weed, *B. pilosa*, which considered as strong nuisance weeds. Therefore, further study is recommended to characterize the mode of actions of the EO from *X. strumarium* as well as determine the biosafety of the integration of this EO as a bioherbicide in agricultural practices as an eco-friendly, green tool.

## Figures and Tables

**Table 1:** Chemical composition of the EO of *X. strumarium* leaves collected from Egypt analyzed by GC-MS.

No	RT ^a^	KI ^b^	KI ^c^	Compound Name	Conc. (%) ^d^
Non-oxygenated Monoterpenoids					
1	29.32	1341	1343.4	1,5-Dimethyltetralin	14.27 ± 0.04
Oxygenated Monoterpenoids					
2	7.93	1346	1341.4	α-Terpinyl acetate	0.56 ± 0.01
3	13.78	1165	1161.9	l-Borneol	6.59 ± 0.02
4	18.44	1285	1266.1	L-bornyl acetate	3.77 ± 0.03
Non-oxygenated sesquiterpenoids					
5	20.86	1374	1319.9	α-Copaene	0.34 ± 0.01
6	22.08	1351	1347.1	α-Cubebene	0.57 ± 0.01
7	22.62	1431	1359.2	β-Copaene	0.39 ± 0.01
8	23.91	1415	1388	Caryophyllene	0.91 ± 0.02
9	26.27	1476	1442.2	γ-Muurolene	0.56 ± 0.01
10	26.49	1482	1447.4	Germacrene-D	0.75 ± 0.01
11	26.86	1492	1455.8	β-Selinene	1.16 ± 0.02
12	26.97	1499	1458.6	β-Guaiene	0.34 ± 0.01
13	28.02	1513	1483.8	β-Cadinene	1.36 ± 0.03
14	28.28	1517	1488.8	Trans-calamenene	0.91 ± 0.01
15	28.64	1474	1497.2	γ-Himachalene	0.29 ± 0.01
16	29.07	1537	1507.5	α-Calacorene	1.34 ± 0.02
Oxygenated Sesquiterpenoids					
17	27.18	1763	1759.3	Aristolone	2.84 ± 0.04
18	29.94	1525	1528.4	9-Methoxycalamenene	0.22 ± 0.01
19	30.04	1578	1531	1,5-Epoxysalvial-4(14)-ene	1.48 ± 0.03
20	30.44	1576	1540.6	Spathulenol	2.49 ± 0.02
21	30.57	1580	1543.8	(-)-Caryophyllene oxide	5.36 ± 0.04
22	30.75	1530	1548.1	Globulol	2.21 ± 0.02
23	31.05	1504	1555.2	Salvial-4(14)-en-1-one	1.83 ± 0.02
24	31.46	1631	1595.2	Aromadendrene oxide-(2)	0.36 ± 0.01
25	31.69	1671	1667.9	Calarene epoxide	3.52 ± 0.03
26	32.04	1537	1579.4	Isolongifolene, 7,8-dehydro-8a-hydroxy-	5.06 ± 0.03
27	32.5	1608	1591.9	(-)-Spathulenol	7.54 ± 0.03
28	32.78	1582	1597.3	Isoaromadendrene epoxide	0.94 ± 0.01
29	33.07	1636	1604.5	Tau-Muurolol	1.76 ± 0.02
30	33.19	1763	1747.6	Aristolene epoxide	3.58 ± 0.03
31	33.49	1654	1615.4	α-Eudesmol	10.60 ± 0.03
32	33.92	1729	1726.1	Murolan-3,9(11)-diene-10-peroxy	0.37 ± 0.01
33	34.11	1548	1547.8	Diepicedrene-1-oxide	1.53 ± 0.02
34	34.85	1729	1739.7	Ledene alcohol	6.46 ± 0.03
35	42.24	1775	1756.3	Furoscrobiculin B	0.30 ± 0.01
36	48.67	2005	2045.9	Isochiapin B	0.53 ± 0.01
37	29.62	1653	1636.8	(+) -γ- Costol	2.80 ± 0.03
Diterpenoids					
38	39.7	2218	2215.6	E-Phytol, acetate	0.49 ± 0.01
39	47.78	2017	2018.4	Kaur-16-ene, (8β,13β)-	0.29 ± 0.01
Oxygenated hydrocarbons					
40	27.86	1512	1517.1	Di-tert-Butylphenol	0.85 ± 0.01
41	40.08	1840	1834.7	2-Pentadecanone, 6,10,14-trimethyl-	1.23 ± 0.02
42	40.55	2566	2561.1	Cis-13,16-Docasadienoic acid	0.52 ± 0.01
43	41.15	2276	2278.9	11,14-Eicosadienoic acid, methyl ester	0.73 ± 0.01

^a^ = Retention time; ^b^ = Kovats retention index on DB-5 column in reference to *n*-alkanes; ^c^ = Experimental Kovates retention index; ^d^ = Values are mean ± standard deviation.

**Table 2:** Correlation matrix (Pearson) between the locations of sampling based on the chemical composition of the EO of both Egyptian *X. strumarium* ecospecies and other ecospecies.

Location	Egypt	Iran-Sistan	Iran-Lurestan	Pakistan	Brazil
Egypt	1	-	-	-	-
Iran-Sistan	−0.11	1	-	-	-
Iran-Lurestan	−0.11	**0.54**	1	-	-
Pakistan	−0.08	0.13	**0.26**	1	-
Brazil	−0.04	−0.01	−0.01	0.02	1

Values in bold are different from zero, with a significance level alpha = 0.05.

**Table 3:** Allelopathic activity of the EO from Egyptian *X. strumarium* on the inhibition of seed germination, root, and shoot growth of *Bidens pilosa* after five days of treatment.

Treatment	Concentration (µL L^−1^)				LSD_0.05_
	250	500	750	1000	
Germination	70.43 ^c^ ± 1.78	82.47 ^b^ ± 2.09	90.77 ^a^ ± 2.30	97.34 ^a^ ± 2.46	7.08
Root	65.42 ^d^ ± 1.65	82.65 ^c^ ± 2.06	90.52 ^b^ ± 2.29	98.45 ^a^ ± 0.52	5.99
Shoot	48.56 ^d^ ± 1.23	73.42 ^c^ ± 1.86	84.52 ^b^ ± 2.14	93.56 ^c^ ± 2.15	6.34

Different letters within each row indicate values significant variation *p* ≤ 0.05.

**Table 4:** Antioxidant activity of the EO from Egyptian *X. strumarium* and ascorbic acid as standard.

Concentration (µL L^−1^)	Scavenging (%) *
500	58.45^a^ ± 1.19
400	52.52^b^ ± 0.79
300	50.20^b^ ± 1.32
200	45.16^c^ ± 1.56
100	38.56^d^ ± 0.71
LSD_0.05_	4.18
IC_50_ µL L^−1^	321.93
IC_50_ Ascorbic acid	35.07

* Values expressed are means ± standard error of three samples. LSD_0.05_: least significant difference at *p* ≤ 0.05. Different letters indicate values significant variation. IC_50_: the amount of sample necessary to decrease the absorbance of DPPH (2,2-Diphenyl-1-picrylhydrazyl) by 50%.
